# Mentalization based treatment of youth on the psychotic spectrum: clinical profiles and outcomes for youth in the ECID

**DOI:** 10.3389/fpsyt.2023.1206511

**Published:** 2023-07-04

**Authors:** Mark Dangerfield, Line Brotnow Decker

**Affiliations:** ^1^Vidal and Barraquer University Institute of Mental Health, Ramon Llull University, Barcelona, Spain; ^2^Yale Child Study Center, Yale University, New Haven, CT, United States

**Keywords:** mentalization-based treatment, psychotic spectrum, adolescence, treatment resistant, AMBIT

## Abstract

**Introduction:**

Early intervention may significantly improve the prognosis associated with psychotic disorders in adulthood.

**Methods:**

The present study examined the acceptability and effectiveness of a standalone intensive, in-home, mentalization-based treatment (MBT) for extremely high-risk, non-help-seeking youth on the psychotic spectrum [Equipo Clínico de Intervención a Domicilio (ECID), Home Intervention Clinical Team].

**Results:**

Despite previously being unable to participate in treatment, more than 90% of youth engaged and those on the psychotic spectrum demonstrated slightly higher engagement than the general high-risk group (95% and 85%, respectively, *X*_1_ = 4.218, *p* = 0.049). Generalized estimating equation (GEE) models revealed no main group effect on the likelihood of reengaging with school over the first 12 months of treatment (*X*_1_ = 1.015, *p* = 0.314) when controlling for the duration of school absenteeism at intake. Overall, the percentage of school engagement rose from 12 to 55 over this period, more than 40% of the total sample experienced clinically reliable change and an additional 50% appeared clinically stable. No statistically significant difference was observed between the groups in the average change in HoNOSCA total severity score (*X*_1_ = 0.249, *p* = 0.618) or the distribution of youth into categories of clinical change during the first year of treatment (*X*_1_ = 0.068, *p* = 0.795).

**Discussion:**

The present findings suggest that a mentalization based intervention may be able to engage extremely high-risk youth in treatment and have clinically meaningful impact on symptom severity and functioning after 12 months.

## Introduction

Psychotic disorders are associated with complex, crippling, and often chronic mental health issues and poor functioning (1, 2). Mounting empirical evidence has revealed that only a small subset of individuals struggling with psychotic states experience an acute onset of symptoms and that as many as 4 in 5 may present with prodromal symptoms for a year or more prior to diagnosis, sometimes labeled *at-risk mental states* (ARMS) or *clinical high risk for psychosis* (CHR-P) (3). Effectively, this period overlaps with adolescence, given that psychotic disorders usually emerge between the ages of 12 and 25 (4). Typically, youth in this group present with difficulties ranging from subtle, subjectively experienced disturbances in mental processes (labeled “Basic Symptoms,” BS) or subthreshold attenuated positive symptoms (APS) to brief limited intermittent psychotic episodes (BIP/BLIP, i.e., with a duration of symptoms of <1 week and with spontaneous remission) or primary schizotypal personality disorder with decline in or chronically low functioning [see Catalan et al. (5)]. However, multiple empirical studies have pointed to the highly comorbid presentations of youth in this group, highlighting the transdiagnostic features associated with high clinical severity in adolescence (5–8).

Previous research has demonstrated that early intervention may significantly improve the prognosis associated with psychotic disorders in adulthood, highlighting the importance of detecting and targeting individuals at heightened risk for developing psychosis during the premorbid or prodromal stages of the assumed clinical continuum (9). Yet, remarking on the large variability in outcomes recorded across studies, recent meta-analyses have shown that only 25% of individuals presenting with ARMS transition to a psychotic disorder after 2–3 years (10). This finding suggests that although psychotic disorders are usually preceded by clinically observable premorbid states, they are not specific to the psychotic spectrum. Interestingly, a majority of the young people identified as being at very high risk and who do not transition to a psychotic disorder do nevertheless continue to struggle with debilitating psychiatric symptoms and poor functioning (11–13), highlighting the importance of preventive interventions targeting this group.

Despite substantial empirical and clinical interest over the past two decades, the evidence base concerning intervention effects with youth on the psychotic spectrum is limited. Several meta-analyses synthesizing data from internationally representative studies examining a range of treatment paradigms (including pharmacological treatment, CBT and family therapy) have found no superior effect of any intervention in the prevention of psychosis (5, 14). Perhaps reflecting this ambiguity, interventions and health care systems vary greatly in terms of their organization (free-standing, integrated into community or hospital services), modes of delivery, focus, and outcomes (15). Initial findings suggest that standalone services were associated with higher acceptability (lower treatment attrition, higher satisfaction, and lower stigmatization), higher effectiveness and higher economic savings. Successful units were also explicitly multidisciplinary, had implemented a clear training protocol and recruited heterogeneous but explicitly high-risk youth [see de Pablo et al. (15) for details].

Taken together, longitudinal studies suggest that <½ of young people at heightened risk of psychosis experience full symptom remission and even fewer regain satisfactory daily functioning and social reinsertion (10) reflecting the “symptom-disability gap” frequently observed in psychiatric research. The authors have highlighted a number of methodological and conceptual issues relating to the measurement of treatment acceptability and effectiveness for youth presenting with at-risk mental states. These include the need for real-world data, studying the impact over time of clinical and functional outcomes of well-defined, transdiagnostic interventions on help-seeking and non-help-seeking youth (i.e., those that don't seek out or accept offers of mental health care), presenting with severe and comorbid symptoms.

Mentalization based treatment was initially developed for adults presenting with borderline personality disorder who struggled to engage in conventional psychotherapy (16). Subsequently, core difficulties with mentalizing (i.e., the ability to make sense of ourselves and others in terms of subjective states and mental processes) have been identified in individuals struggling with mental health issues as seemingly diverse as eating disorders, autism spectrum disorder, psychosis and a range of personality disorders across ages and clinical settings, leading to its conceptualization as a transdiagnostic treatment model (17–20). Across diagnostic categories, difficulties with mentalizing are associated with greater symptom severity and poorer functioning, and mounting evidence suggests focused interventions may impact the quality and sturdiness of the individual's mentalizing capacity as a mediating factor in symptom remission (21, 22). MBT may be particularly effective for individuals presenting with more severe clinical symptoms (23). Additional research is required to establish the precise causal mechanisms at play and the effectiveness of MBT over other treatment options in naturalistic settings (17).

Research into the role of mentalizing in the development of psychotic spectrum disorders is budding and some empirical findings suggest that mentalizing may be a protective factor in the context of at-risk mental states [e.g., (24, 25)]. Conversely, deficits in social cognition are common among youth presenting with at-risk mental states (26) although specific causal mechanisms linking these difficulties with the transition to psychotic disorders have yet to be established [see Debbané and Toffel (27) for a review of findings]. One study found that reflective functioning predicted specific pre-clinical psychotic symptoms as well as a heightened likelihood of transitioning to a psychotic disorder among youth presenting with ARMS (24). Mentalizing may thus appear as a protective factor at different stages of the continuum of psychotic states (28). As yet, it is unclear whether these findings are best explained by characteristics specific to the development of psychotic symptoms *per se* or due, at least in part, to the psychiatric comorbidities that frequently exist alongside them, including personality disorders (6, 8, 29). A mentalization-based approach to at-risk mental states or psychotic disorders could therefore mechanistically either target psychosis-specific phenomena or a general psychopathology factor assumed to be present across symptom clusters.

The goal of mentalization-based interventions is to facilitate the emergence or solidify the young person's capacity for mentalizing, namely as a resource in potentially overwhelming situations. The intervention is explicitly relational, initially aiming to establish an increasingly trusting and secure relationship between the young person and the clinician which mimics the developmental context within which mentalizing usually develops (16). Specifically, recent developments in the understanding of mentalizing have highlighted the importance of *epistemic trust* in this context, meaning trust in the authenticity of interpersonally transmitted knowledge (30). Following mentalizing theory, distrust in the therapeutic relationship can be understood as an adaptive response to living in threatening and unsupportive social contexts (31), but will nonetheless hamper the intergenerational transmission of sociocultural knowledge. Consistent with this conceptual development and recent empirical findings suggesting that the impact of mentalization-based interventions may be gauged at different levels of analysis, central thinkers in the MBT tradition have highlighted the importance of examining outcomes relating to the therapeutic relationship (reflecting changes in epistemic trust), clinical profiles, and real-life functioning (32).

MBT interventions are manualized and characterized by their focus on coherence, consistency and continuity (33). Therapeutic interactions are designed to maintain attention and emotion regulation at levels that allow for increased affective awareness and perspective taking without becoming overwhelmed [e.g., (34)]. Attention is usually focused on real-life situations or here-and-now interactions between the young person and the clinician, highlighting the intervention's explicit focus on experiential, individualized learning.

In terms of their mode of delivery, mentalization-based treatment interventions for young people usually incorporate working with caregivers and the family group as well as focusing explicitly on the youth's social and academic functioning. This is especially prominent in models such as the Adaptive Mentalization Based Integrative Treatment (AMBIT) which targets youth with particularly severe or comorbid presentations, working in multidisciplinary teams across developmental arenas to engage otherwise non-help-seeking youth (35–37). Teams working within the AMBIT model have shown positive outcomes across a range of services (38, 39).

Some initial clinical applications of mentalization-based treatment principles with youth on the psychotic spectrum have focused on its adaptation to the assumed stages of the psychotic continuum [e.g., (27)], noting particularly the relevance of targeting the young person's social functioning (40). There is empirical evidence supporting the effectiveness of Mentalization-Based Treatment for Adolescents (MBT-A) in reducing self-harm and depression (41), but empirical evidence of the feasibility and effectiveness of mentalization-based interventions with youth on the psychotic spectrum is still pending.

The Equipo Clínico de Intervención a Domicilio (ECID, Home Intervention Clinical Team) is an intensive, in-home, mentalization-based treatment program targeting extremely high-risk and non-help-seeking adolescents in Barcelona, Spain. Their risk-profile can be described on multiple levels, ranging from their typically high number of predisposing factors (e.g., transgenerational trauma, social marginalization, poverty), extremely severe and comorbid diagnostic presentations (severe anxiety and mood disorders, complex eating disorders, psychotic spectrum disorders, and personality disorders) significant functional deficits (including chronic school absenteeism and criminal justice involvement) and an explicitly non help-seeking stance excluding them from participating in other community- or inpatient treatment to which they have all previously been referred (42–44).

The fact that they have not previously been successfully engaged by mental health programs despite their clinical acuity is a unifying but highly complex trait shared by the young people in the ECID. At the diagnostic and symptomatic surface, the adolescents present with highly diverse profiles, ranging from those deeply withdrawn teenagers who have not left their room for months and years, appearing mute and disconnected, to those high-intensity youth who engage in risky behaviors with and without peers outside of the home and school environments. This notwithstanding, our clinical experience, based on in-depth structured and observational assessments, tells us that the young people we work with all have had intensely painful experiences in their primary relationships, leaving them with a feeling of emotional isolation, epistemic mistrust, hopelessness and hypervigilance in the face of relational intimacy. Youth with severe and complex psychopathological symptoms and poor functioning, whose adaptive responses to experiences of relational trauma, exclusion and marginalization, have understandably left them reluctant to trust in others, especially mental health services. They present multiple and often overlapping needs, as well as significant high risk. However, accessing and using mainstream mental health services is particularly challenging. Many of the caregivers involved in the ECID have similar relational histories and expectations, which we assume relates to the disorganization, rejection and mistrust we often see impacting the family system as a whole. Many of the families involved in the ECID also belong to historically or systematically marginalized or oppressed communities with lived experiences of transgressions at the hands of “helpers” (45, 46). Our clinical experiences with the non-help-seeking stance of the young people we see paired with the growing empirical literature on epistemic trust provide a foundational conceptual framework for our approach to treatment, in line with the evolving causal model underpinning MBT (47).

The principal goal of the ECID is to engage the adolescent in the process of resuming a life project, which includes care for their mental disorders, re-engagement with school and scaffolding existing relationships, while managing the significant risks present in their relational contexts. To this end, each adolescent is assigned an individual clinician (clinical psychologist, psychiatrist, social worker, or mental health nurse) whose goal is to facilitate a relational experience that allows the development of epistemic trust that can be generalized to the wider relational and social network around the young person (44). The aim is to offer a relationship in which the young person can revisit the psychological developmental process that leads to a sense of agency and trust, which in turn facilitates mentalization (48, 49). This can only happen through highly individualized interactions, truly meeting the young person “where they are” both physically (in their room, at the park) and emotionally, focusing particularly on validating the young person's life experiences and suffering without triggering overwhelming affective states or stigmatization.

The ECID team also works closely with the primary caregivers and other important people in the young person's life, focusing on solidifying their own mentalizing capacity (curiosity, openness, perspective taking) and fostering supportive relationships inside and outside the family (50). A central principle guiding this work is an outreach approach that takes the therapeutic perspective to the young person and family's daily lives, focusing on adapting to their attachment capacities. Throughout their time with the young person, the clinician aims to model openness, not-knowing, curiosity and safety in seeking support from others. In line with the AMBIT model and the core tenet of MBT that mentalizing begets mentalizing, clinicians work in multidisciplinary teams specifically organized to provide broad clinical expertise and a supportive environment which facilitates the clinician's ability to mentalize the young person and regulate their own emotional responses to the high-intensity therapeutic work (36, 37). The ECID offers Mentalization-Based Treatment for Adolescents (MBT-A) delivered in an AMBIT framework, which implies a mentalization based approach to support not only our work with the adolescents and their families, but also to identify and address the difficulties in mentalizing that inevitably occur when working with our teams, working with the wider network of services and professionals involved in the young person's and family's care and in the process of learning at work.

In summary, psychotic disorders are usually preceded by a hypothesized critical prodromal period of 2–5 years marked by a plethora of difficulties conceptualized as a continuum of symptoms (at risk mental states, ARMS). However, most youth presenting with ARMS do not transition to a psychotic disorder and many present with non-psychotic comorbid states and intractable functional difficulties. Despite mounting empirical interest, treatment programs explicitly targeting the prevention of psychotic disorders have demonstrated only moderate effectiveness and a majority of those presenting with ARMS in adolescence or early adulthood do not go on to experience symptomatic or functional remission. In light of this, some have argued in favor of the reconceptualization of ARMS in terms of pluripotential states for a range of disorders, requiring specialized but transdiagnostic or transsyndromal long-term care (8). Mentalization-based treatment appears among these interventions, and there is increasing evidence of its effectiveness cutting across diagnostic categories. As yet, no conclusive empirical findings have demonstrated its feasibility or effectiveness with youth presenting with at-risk mental states. The ECID is an intensive, in-home, mentalization-based treatment program targeting non-help-seeking youth with severe and complex symptoms and poor functioning.

The present study will examine the clinical profiles and treatment outcomes of youth on and off the psychotic spectrum enrolled in the ECID program. To this end, we will describe demographic and clinical differences at intake as well as differences in engagement and outcomes for youth on and off the psychotic spectrum receiving intensive, in-home, mentalization-based treatment. We expect that a meaningful proportion of the high-risk youth enrolled in the ECID will meet criteria for being on the psychotic spectrum. Clinical experiences with this group lead us to expect that youth with such high-risk mental states are able to engage in treatment at similar rates to other youth. Our expectation is that they will show similar rates of clinical improvement overall.

## Materials and methods

### Participants

Adolescents deemed eligible for treatment in the ECID are between 11 and 18 years old at intake, present with severe mental health symptoms and poor functioning, and have not been able to engage in previously initiated community-based or hospital treatment (labeled “non-help-seeking”). No other psychopathology exclusion criteria apply. Treatment duration varies naturally as a function of client needs and data was analyzed as a function of intention to treat, subject to availability.

### Intervention

The ECID operates as a standalone mental health care unit within the Catalan health care system (CatSalut), occupying a unique position within the continuum of care. It offers a 2-year, mentalization-based intervention provided by a multidisciplinary team of clinicians all certified in mentalization-based treatment for adolescents (MBT-A) and AMBIT. In practice, the intervention consists of implementing the MBT-A manual (51) within an AMBIT framework. Clinicians interact with young people and their family members according to the interventions described in MBT-A while working in transdisciplinary teams outside of regular outpatient or inpatient settings. The ECID intervention incorporates a number of standardized elements such as structured, regular assessments (carried out by two clinicians and discussed with the multidisciplinary team), therapeutic sessions with the young person, parent and family, as well as case management, including reconnecting with appropriate medical and academic resources. The clinicians usually meet with the young person and family on a weekly basis (separate clinicians work with the young person and the family) and coordinate interactions with other relevant collaborators, including teachers. The ECID team meets weekly to discuss cases using “Thinking Together,” a structured AMBIT tool for supporting a mentalization-based approach to helping conversations, with an emphasis on attending to the mind of the clinician and supporting his own mentalizaing (37), and also receives fortnightly group supervision by certified MBT-A and AMBIT supervisors.

### Procedure

The present analysis is based on data collected in the context of ordinary clinical activities in the ECID. Demographic and clinical background data is gathered by the clinician within the first few weeks of treatment and summarizes information from conversations with the family and young person and available medical charts. Standardized clinical assessments of the youth (such as the HoNOSCA) are performed as early as possible in the treatment, depending on the young person's ability to interact with the clinician and thereafter repeated at 6-month intervals throughout treatment. The data is entered into a secure, digital system by the clinician upon collection and stored in accordance with Spanish government guidelines.

### Measures

*Psychotic Spectrum*. Youth categorized on the psychotic spectrum (PS) either presented with At Risk Mental States (ARMS) or a current psychotic disorder. They are assessed upon enrollment in the ECID by a licensed clinical psychologist and/or psychiatrist who determines whether diagnostic criteria have been met for any psychiatric disorders according to the International Statistical Classification of Diseases and Related Health Problems [ICD, 11th ed., (52)]. In conjunction with this formal assessment, the clinical team—formally trained in the use of the scale—determines whether the youth presents with current at-risk mental states, using the Spanish language version of the 15-item ERIraos Early recognition inventory Check List (53, 54). The questionnaire includes items capturing a range of subjectively experienced differences in perception or cognitive, affective, motor, somatic functioning [e.g., suspiciousness, thought disturbances (such as delusions or hallucinations), derealization, depressive mood, and novel experience of bodily functioning] and observable behaviors (e.g., self-neglect, social withdrawal, altered psychomotor tempo, and reduced performance in school or work). Previous literature has established a 12-point threshold as a clinical cut-off to distinguish those at higher risk for transitioning to a clinical diagnosis (54). The instrument has demonstrated adequate psychometric qualities (55, 56).

*Treatment engagement* in the ECID was defined as an adolescent explicitly accepting and following through with their commitment to meet with the ECID professional with the proposed frequency of sessions over the course of the treatment relationship. Given the inherent variability of the individualized treatment plan for each young person, this determination was made by the clinician in collaboration with the clinical team.

*The Health of the Nation Outcome Scales for Children and Adolescents (HoNOSCA)* is a widely implemented, brief clinician-reported measure of mental health symptoms and functioning in children and adolescents. It has demonstrated adequate psychometric properties in clinical populations across clinical settings and levels of severity. The scale comprises 15 items, each rated on a scale of 0 (“no problems”) to 4 (“severe problems”). The score is determined by the clinician based on an interview with the young person. A total severity score is computed based on 13 core items, with a possible range of 0–52.

Interpreting the total symptom score as an indicator of clinical severity requires careful consideration as it is not understood to represent a single latent construct of psychopathology. An individual can therefore be labeled clinically severe with an elevated score on only one or a few items even if their total score is low, potentially making the total score an inadequate reflection of severity. Analyzing individual item scores thus yields a more accurate picture of severity but opens the door to a high number of potential analyses. Merging the need for a single overarching measure of severity and usefulness of examining individual items, researchers have suggested tallying the rates of items receiving elevated scores [see for example (57–59)]. In line with these recommendations, the present study will categorize individuals according to the following scale: “subclinical” (no scores of 2 or higher), “mild” (one or more scores of 2), “moderately severe” (scoring 3 on any item), and “very severe” (scoring 3 or higher on two or more items). Clinical change over time on the HoNOSCA has typically been described either in terms of difference scores (“statistically significant change”), in terms of the percentage of youth whose difference in total score reliably increased, decreased or remained unchanged, or in terms of the proportion of youth transitioning from a dysfunctional to a non-clinical status (“clinically significant change”). The present study will report differences in mean total scores for the two groups as well as the proportion of each group demonstrating clinically reliable improvement, stability or worsening of their symptoms, defined as a change of 8 or more points in either direction after 6 and 12 months of treatment.

*School reengagement*. School reengagement was defined as having initiated or returned to an educational program (academic or vocational training), operationalized as attending the planned activity minimum of 2 or 3 times per week (or 9–14 days per month). The youth's status was recorded at baseline and at 3-month intervals thereafter until the end of treatment.

### Data analyses

Data were analyzed using the IBM Statistical Package for the Social Sciences (SPSS), version 28. Descriptive and inferential analyses were performed to examine characteristics of the full sample at baseline as well as differences between the two groups. Variables indicating statistically significant differences at intake were included as covariates in subsequent testing of group differences in treatment outcomes.

Chi squared tests were performed to estimate differences in the likelihood of the young person actively engaging in treatment and remaining in treatment beyond 12 months. We performed a Student's *t*-test to determine group differences in average treatment duration.

To account for the non-independence of the repeated measures of the HoNOSCA (at 6 and 12 months) and school reengagement (at 3, 6, 9, and 12 months), the data were analyzed by fitting a generalized estimating equation model (GEE) (60). GEE is an increasingly utilized multilevel regression technique that adjusts standard errors for correlated data (such as in longitudinal designs) and avoids issues pertaining to multiple comparisons (61). It allows for the examination of non-normal distributions of the independent and dependent variables, including binomial distributions. A working correlation structure is determined *a priori*, defining the assumed (theoretical) relationship between the repeated measures. As is frequently the case for longitudinal data, the current analyses were performed using an autoregressive correlation structure (AR-1), which assumes stronger correlations for observations closer together in time. In line with current recommendations for models containing dichotomous variables, a generalized score statistic (Chi squared) was calculated and reported for each model. Test assumptions were examined and reported where relevant.

## Results

Between November 2017 and February 2023 131 adolescents aged 11–18 years (mean 14.9 years, 54% male) and their families were enrolled in the ECID. The average duration of treatment for all enrolled participants at the time of the present analysis was 19 months (*SD* = 11.9 months). 7.1% of youth left the program in the first 6 months of treatment. It is not possible to distinguish between those who successfully transitioned to a lower level of mental health care and those who simply discontinued treatment.

### Demographic and clinical profiles at baseline

The mean clinical severity of the full sample at baseline as measured by the HoNOSCA was 22.4, with scores ranging from 9 to 37. Of the 91 youth with available scores on all HoNOSCA items at baseline, all but two (98%) were categorized as being “very severe” (i.e., scored 3 or higher on two or more items). Eighty eight percent of the youth enrolled in the ECID presented with chronic school absenteeism at the start of treatment ranging in duration from 0 to 36 months (*m* = 15.3).

Forty seven percent of the full sample was determined either to be presenting with at risk mental states (ARMS) or currently meeting criteria for a psychotic disorder (henceforth labeled Psychotic Spectrum, PS). The psychotic spectrum and non-psychotic spectrum groups were indistinguishable in their age and gender distributions (*t*_119_ = 1.04, *p* = 0.30 and *X*_1_ = 0.241, *p* = 0.377) as well as in their clinical severity at baseline as measured by the HoNOSCA total score (*t*_91_ = 1.671, *p* = 0.098). Although matched in terms of the likelihood of attending school at intake (*X*_1_ = 1.547, *p* = 0.274), youth on the psychotic spectrum had on average been absent from school 60% longer than the general high-risk group at the start of treatment (*t*_120_ = 4.26, *p* < 0.001). Duration of school absenteeism at intake was therefore included as a covariate in the analysis of school reengagement. See Table 1 for details.

**Table 1:** Demographic and clinical profiles at baseline by group.

	***n***	**Gender**	**Mean age (*SD*)**	**HoNOSCA total score (*M*)**	**School absence (%)**	**School absence months (*M*)**
PS	62	56% male	15.0 (1.4)	23.4	91.5	19.0
Non-PS	69	52% male	14.8 (1.6)	21.7	84.1	11.7

School absence (%) = percentage of each group considered chronically absent at intake.

School absence months (M) = average duration of school absence at intake by group.

### Outcomes

#### Engagement with ECID

Ninety-five percent of youth on the psychotic spectrum and 85% of youth not on the psychotic spectrum engaged in treatment. This difference is statistically significant (*X*_1_ = 4.218, *p* = 0.049). For those already discharged from treatment, youth on the psychotic spectrum are more likely to remain in treatment past 12 months (94 and 80% for PS and non-PS respectively, *X*_1_ = 5.276, *p* = 0.024) and youth in this group also remain in active treatment longer on average (25 vs. 20 months for PS and non-PS respectively, *t*_82_ = 2.382, *p* < 0.020).

#### Reengagement with school

School reengagement was statistically significantly correlated between all time-points for the full sample (see Table 2). Data was therefore analyzed by fitting a logistic generalized estimating equation (GEE) model, assuming an autoregressive (AR-1) correlation structure with duration of school absenteeism at intake and time as continuous covariates. The interaction effects of group with school absenteeism and time with school absenteeism were also examined.

**Table 2:** Pearson correlations between school reengagement rates at 0, 3, 6, 9, and 12 months with percentage engaged and sample size.

	**3 mo**	**6 mo**	**9 mo**	**12 mo**	**% engaged**	***N***
Baseline	0.846^**^	0.534^**^	0.376^**^	0.264^*^	12.3	122
3 mo		0.620^**^	0.436^**^	0.312^**^	16.8	131
6 mo			0.448^**^	0.449^**^	29.2	113
9 mo				0.533^**^	57.4	101
12 mo					54.9	91

^**^p < 0.001.

^*^p < 0.05.

**Figure 1:**
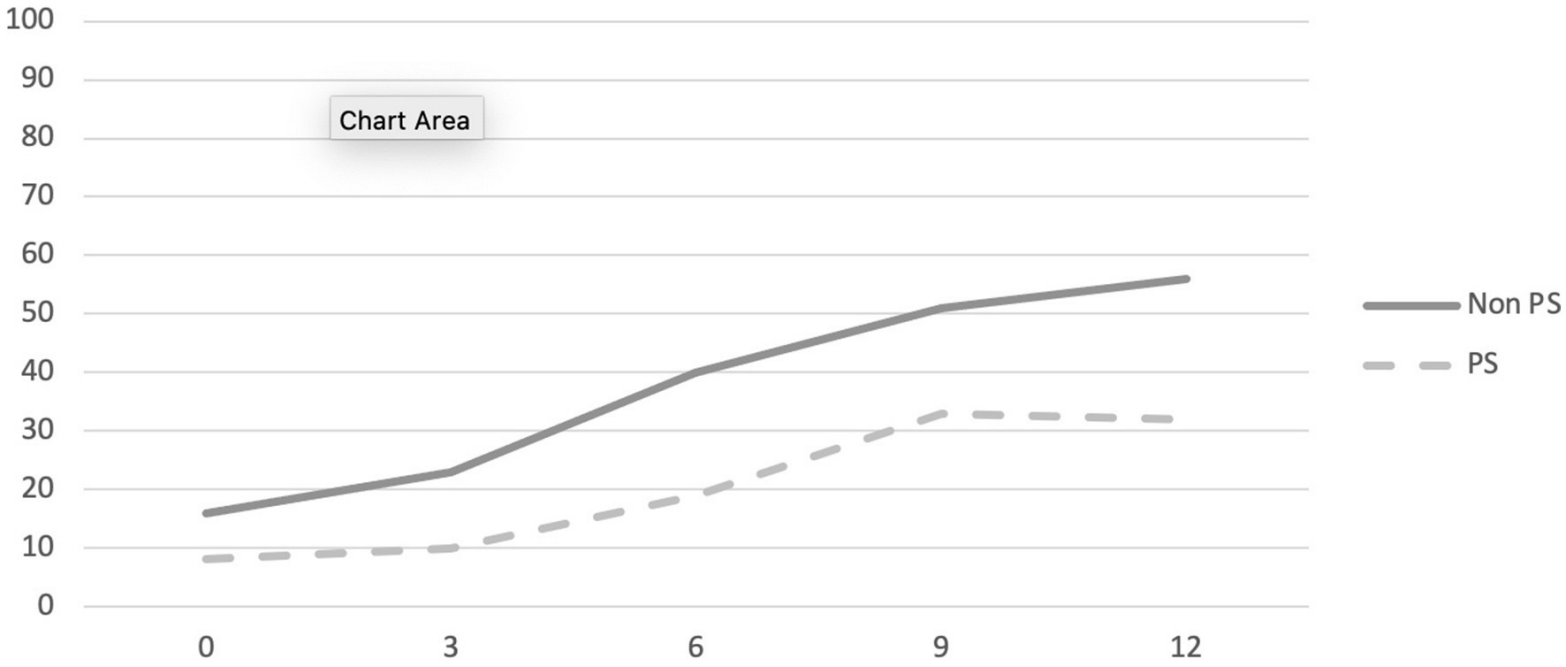
Percentage of sample engaged in school at 3, 6, 9, and 12 months by group.

The likelihood of school engagement for the whole sample increased from 12 to 55% over the course of the 1st year of treatment. Results indicate a main effect of duration of school absenteeism (*X*_1_ = 15.371, *p* < 0.001) but not of group (*X*_1_ = 1.015, *p* = 0.314) or time (*X*_1_ = 0.003, *p* = 0.959). The interaction effect of school absenteeism with time was significant (*X*_1_ = 18.174, *p* < 0.001) whereas that of group and school absenteeism was not (*X*_1_ = 0.037, *p* = 0.848). For the sample as a whole, the timeline for returning to school differs as a function of the duration of school absenteeism at intake. No statistically significant differences were observed between the two groups in terms of their return to school over 12 months. See Figure 1 and Table 3 for complete details.

**Table 3:** GEE marginal model parameters estimating association between study group, school absences and time with likelihood of engaging with school at 0, 3, 6, 9, and 12 months.

	**B**	**SE**	**95% CI**
PS	−0.589	0.5917	−1.749-0.571
School absence	0.295	0.0718	0.155-0.436
Time	0.007	0.1266	−0.242-0.255
PS*School absence	0.009	0.0422	−0.073-0.092
PS*Time	−0.052	0.013	−0.077- −0.026

#### Change in HoNOSCA scores at 6 and 12 months

The HoNOSCA total score was statistically significantly correlated between all time-points for the full sample (see Table 4). Data was therefore analyzed by fitting a linear generalized estimating equation (GEE) model, assuming an autoregressive (AR-1) correlation structure.

**Table 4:** Pearson correlations among month 0, 6, and 12 HoNOSCA total score with means, SD, skewness, kurtosis, and sample size.

	**6 mo**.	**12 mo**.	**M**	**SD**	**Skew**	**Kurtosis**	***N***
Baseline	0.610^**^	0.303^*^	22.4	5.85	0.124	−0.561	121
6 mo.		0.525^**^	19.7	6.54	0.203	0.092	92
12 mo.			17.0	5.62	0.358	−0.160	58

^**^p < 0.001.

^*^p < 0.05.

The average HoNOSCA total severity score dropped by 3.3 and 3.5 points in the first 6 months for the PS and non-PS groups respectively and by 6.3 and 6.7 points in the first 12 months. Results of the marginal effect model (GEE) indicate no main effect of group [*X*_1_ = 0.249, *p* = 0.618, β = −3.307 *S.E*. = 6.62 (*CI* 95% = −16.286–9.672)], suggesting there are no differences in HoNOSCA total severity score changes between the two groups over time.

**Figure 2:**
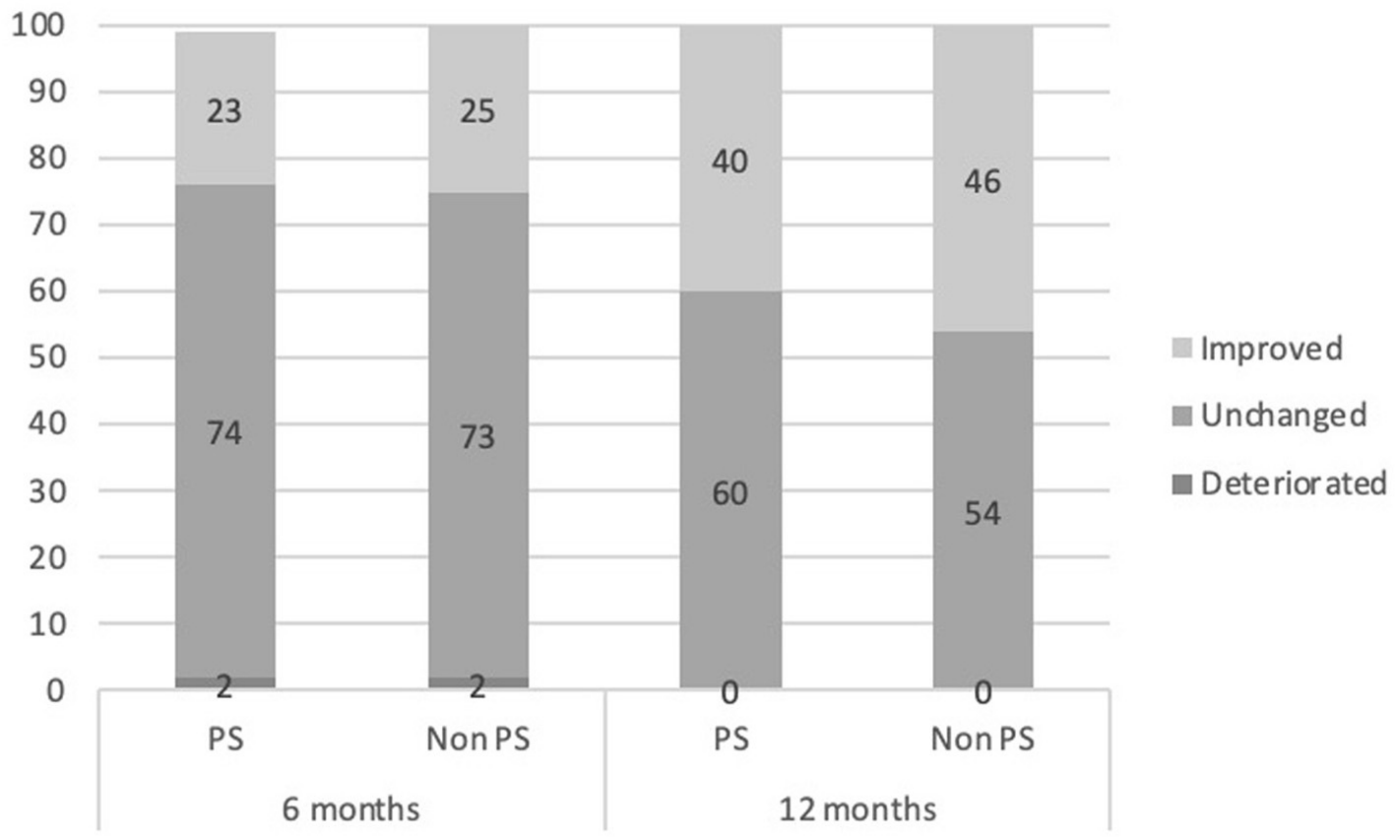
Percentage clinically reliable change by category for both study groups comparing baseline to 6 and 12 months of treatment.

Reliable change is defined as 8 points or more, making a decrease by 8 or more points a reliable improvement and an increase by the same amount a reliable deterioration. An ordinal GEE model was fitted to estimate the association between group and the distribution of youth into categories of change at six and 12 months. Results indicate no main effect of group, suggesting there is no statistically significant difference between the two groups in terms of the distribution of youth into categories of change [*X*_1_ = 0.068, *p* = 0.795, β = −0.099 *S.E*. = 0.3761 (*CI* 95% = −0.638–0.836)]. See Figure 2 for details.

## Discussion

The present study compared the demographic and clinical profiles of youth on the psychotic spectrum entering intensive, in-home mentalization-based treatment as well as their treatment outcomes over the first 12 months of treatment with those of youth presenting with a generally high-risk (non-psychotic) profile. To our knowledge, this is the first empirical study examining MBT treatment outcomes for high-risk, non-help-seeking youth on and off the psychotic spectrum.

The feasibility and acceptability of the intervention were examined by looking at the proportion of the young people in each group engaging in treatment as well as their treatment duration. Despite previously not having been successfully engaged by community-based and inpatient treatment programs, youth in both groups were overwhelmingly willing to participate in the intervention. Youth on the psychotic spectrum were statistically significantly more likely to engage (95% vs. 85 for the non-PS group) and remained in treatment longer on average. The low levels of drop-out from the ECID intervention aligns with previous literature demonstrating the high acceptability of MBT for high-risk groups (62).

In line with previous research (5, 6, 8, 29) and study hypotheses, the two study groups present with similarly high and complex symptomatology at intake, most struggling with comorbid psychiatric conditions. Youth on and off the psychotic spectrum experience similar rates of symptom reduction over the course of the 1st year of treatment, with more than half appearing clinically stable and four in 10 demonstrating clinically relevant improvement according to the predetermined criteria of the HoNOSCA scales after 12 months. Additional treatment studies are required to contextualize this finding, although it appears significant in light of previous research (15).

Nearly all the adolescents enrolled in the ECID have been absent from school for an extended period at the outset of treatment, many for a year or more. In addition to the obvious detriment to their academic progress, this represents the loss of a key developmental arena in adolescence. School engagement was therefore examined as a core indicator of daily functioning for the group of extremely high-risk youth in the ECID. Our results suggest that more than half youth in the ECID return to school during the 1st year of treatment. In line with previous findings suggesting that youth on the psychotic spectrum present with lower functioning that other high-risk youth, these young people had been absent from school significantly longer at intake than the comparison group. Results indicate that the duration of school absenteeism prior to enrolment predicts the rate of return for each group. We found no main effect of being on the psychotic spectrum, suggesting this distinction is not of primary relevance for this outcome.

A number of clinical and empirical findings can help shed light on the present findings. The analysis of treatment engagement, symptom severity and school functioning suggest that the youth on the psychotic spectrum are nearly indistinguishable from those high-risk adolescents not presenting with at-risk mental states. This is consistent with findings from empirical literature suggesting that ARMS may co-occur with a range of non-psychotic symptoms, yielding highly complex, comorbid presentations with variable levels of severity (5–8). The only statistically significant differences between the two groups in the current study was the ability of youth on the psychotic spectrum to engage in treatment (higher rates of engagement, longer treatment duration) and the earlier onset and duration of school absenteeism at intake (60% longer than those not on the psychotic spectrum). The latter finding may indicate more entrenched functional difficulties among these youth, which is also in line with previous findings. However, both groups are equally likely to experience chronic school absenteeism at intake and reengage at similar rates over the first 12 months of treatment. Taken together, these findings appear to align with previous studies revealing a transdiagnostic effect of treatment for high-risk youth independently of the presence of ARMS (8).

Previous literature has found that mentalization-based interventions can be effective with very high-risk young people, cutting across conventional diagnostic categories. The present findings lend further empirical support to this notion, demonstrating very high levels of treatment acceptability and a substantial proportion of youth experiencing clinically relevant improvement on broad indicators of clinical severity and functioning. Previous literature has suggested aspects of mentalization-based treatment that may be particularly relevant and effective for high-risk youth with severe and complex clinical presentations [see Debbané et al. (28) for a review]. Like the ECID, mentalization-based interventions typically highlight the importance of establishing a strong working alliance by modeling an explicitly non-expert, not-knowing stance and going at the pace of the young person. This may be of particular relevance for youth on the psychotic spectrum whose confusion and suspiciousness may make the establishment of a trusting relationship even more difficult. But the presence of painful early experiences within primary relationships which is present across the sample of non-help-seeking youth seen in the ECID may also account for this assumed effect. The high level of engagement demonstrated by this historically difficult to engage group seems to strengthen the notion that MBT can increase epistemic trust and the chronologically ensuing improvement of symptom severity and educational attainment lend additional empirical support to the prevailing conceptual models of causal mechanisms associated with MBT (32).

Overall, the present findings lend initial empirical support to the notion that mentalization-based treatment may be acceptable and effective for youth presenting with at-risk mental states (ARMS) or psychotic disorders. The study responds to previously identified gaps in the empirical literature by examining real-life data collected in the context of ordinary clinical services provided to a highly heterogeneous group of high-risk adolescents who have not previously successfully engaged in mental health treatment. The latter feature may be particularly relevant, as previous research on treatment outcomes for youth on the psychotic spectrum has focused—naturally—on help-seeking individuals (8). The relevance and generalizability of the findings is further strengthened by the inclusion of a range of outcomes, with a focus on broad clinical indicators with established criteria for reliable clinical change and objectively observable functional markers.

These strengths notwithstanding, the present findings should be interpreted with caution and in light of several potential limitations. First, the present study did not include a conventional measure of treatment attrition. In the ECID, youth may remain in treatment despite not being personally engaged as long as their caregivers are perceived to benefit from it. A very small minority of youth never engage but nonetheless remain connected with the program and may still draw benefit from it. Treatment duration is highly variable both for those young people who engage and those who do not. Further analysis is required to establish whether any particular subgroup of youth is likely not to engage and to leave treatment without a suitable further treatment plan. Second, although the measures included in the present study are empirically validated, all outcomes are clinician-rated or -reported. Given the substantial discrepancies typically found between reporters of symptom severity and functioning in adolescence (63), aggregating scores from the young person, parent, and clinician may yield more accurate depictions of the adolescent's clinical functioning. Third, previous literature has identified clinically meaningful subgroups of youth on the psychotic spectrum, ranging in symptom profile and severity as well as their likelihood of progressing toward a psychotic disorder. The present study was limited by its binary definition of psychotic spectrum difficulties and low number of youth with a confirmed psychotic disorder. Future research should investigate whether intensive, in-home mentalization-based treatment has comparable effects on youth across these subgroups.

In summary, youth with at-risk mental states are likely to appear alongside other high-risk youth in generalized mental health care settings, often presenting with significant non-psychotic comorbid psychiatric symptoms and risk factors in addition to ARMS. A transdiagnostic, mentalization-based, person-centered intervention program such as the ECID, targeting youth presenting with a general high- risk, non-help-seeking profile may be an appropriate and effective treatment option also for youth on the psychotic spectrum. This appears in line with authors suggesting the wider implementation of general at-risk clinics for early stage pluripotential syndromes.

## Data availability statement

The raw data supporting the conclusions of this article will be made available by the authors, without undue reservation.

## Ethics statement

The studies involving human participants were reviewed and approved by the Research Ethics Committee of the Vidal and Barraquer University Institute of Mental Health, Ramon Llull University (URL). Parents or legal tutors of the adolescents signed a written informed consent that includes clinical aspects and information about the fact that anonymous clinical, age and gender data would be used for research purposes.

## Author contributions

MD and LB contributed to the conception and design of the study. LB organized the database, performed the statistical analyses, and wrote the first draft of the paper. MD wrote sections of the paper. Both authors contributed to manuscript revision and approved the submitted version.
